# A Simple *in vivo* Assay Using Amphipods for the Evaluation of Potential Biocompatible Metal-Organic Frameworks

**DOI:** 10.3389/fbioe.2021.584115

**Published:** 2021-02-01

**Authors:** Ines Sifaoui, Idaira Pacheco-Fernández, José E. Piñero, Verónica Pino, Jacob Lorenzo-Morales

**Affiliations:** ^1^Instituto Universitario de Enfermedades Tropicales y Salud Pública de Canarias, Universidad de La Laguna, San Cristóbal de La Laguna, Spain; ^2^Departamento de Obstetricia, Ginecología, Pediatría, Medicina Preventiva y Salud Pública, Toxicología, Medicina Legal y Forense y Parasitología, Universidad de La Laguna, San Cristóbal de La Laguna, Spain; ^3^Red de Investigación Cooperativa en Enfermedades Tropicales, Madrid, Spain; ^4^Laboratorio de Materiales para Analísis Químico (MAT4LL), Departamento de Química, Unidad Departamental de Química Analítica, Universidad de La Laguna, San Cristóbal de La Laguna, Spain

**Keywords:** metal-organic frameworks, cytotoxicity, amphipods, macrophage, *in vivo*

## Abstract

In this study, the application of amphipods *in vivo* assays was evaluated. The main aim of this work was to check the potential use of this model in biocompatibility assessments of metal-organic frameworks (MOFs). Hence, six different MOFs were synthesized and the *in vitro* and *ex vivo* cytotoxicity was first assessed using a colorimetric assay and a macrophage cell line. Obtained results were compared to validate the *in vivo* toxicity tests carried out using amphipods and increasing concentrations of the different MOFs. Amphipods do not require the need of ethics approval and also are less expensive to keep than conventional *in vivo* models, showing its potential as a fast and reliable platform in toxicity studies. The obtained results showed that the amphipods based-assay was simple, easy to replicate and yielded toxicity data corresponding to the type of MOFs tested. In addition, it was observed that only CIM-80(Al) and CIM-84(Zr) did not show any toxicity to the animals at the different tested concentrations. Therefore, the developed *in vivo* model could be applied as a high-throughput toxicity screening method to evaluate the toxicity of numerous materials, chemicals and therapeutic agents among others.

## Introduction

Metal-organic frameworks (MOFs) are a group of crystalline materials formed by the combination of two building units: metallic clusters and organic ligands (Kong et al., 2013). They are assembled by strong coordination bonds forming a highly ordered three dimensional network, which provides an impressive porosity to the material with the highest surface areas known (Kalmutzki et al., 2018). Furthermore, they present other interesting properties, such as synthetic versatility, and chemical, mechanical and thermal stability, which can be tuned by designing and selecting the adequate building units from the broad available spectrum (Kalmutzki et al., 2018). All these features make MOFs attractive materials for many applications in different scientific fields, including catalysis, gas storage, separation, sensing, energy, and even biomedicine (Wang et al., 2018; Yuan et al., 2018; Gutiérrez-Serpa et al., 2019; Wang and Astruc, 2019; Dou et al., 2020; Yang and Yang, 2020; Zhou et al., 2020).

Due to unceasing advances within MOFs development and the increasing number of applications reported, the concern on the toxicity of MOFs have emerged in the last years (Ye et al., 2019). In this sense, most guidelines dealing with the sustainability of MOFs focus on their composition: nature of metal cluster and organic linker, and on the synthetic conditions, such as type of solvent, mode of synthesis, energy consumption, and costs (Kumar et al., 2020). However, the discussion or the study of the MOF toxicity itself is barely found in the literature and requires a special improvement given the release of metal ions during MOF degradation and the nano-size of most MOFs, which may make them highly toxic due to their easily penetration in tissues and cell membranes (Kumar et al., 2019).

There are several studies in the literature that evaluate the cytotoxicity of MOFs using well-known eukaryotic cell lines (Simon-Yarza et al., 2018). Most of these approaches are based on the incubation of the cell lines with MOFs to be tested (Zhu et al., 2020). However, these approaches are not always ideal for this type of materials since they are water-insoluble solids that tend to sediment in the bottom of the well plate and are too large causing physical damage more than cellular toxicity (Ren et al., 2014).

Despite the relative success of these assays when evaluating the cytotoxicity of MOFs, they should mimic *in vivo* conditions since the lack of *in vitro* toxicity does not mean the material is totally safe and biocompatible (Simon-Yarza et al., 2018). Therefore, it is important the development of reliable *ex vivo* or *in vivo* assays for the assessment of the toxicity of these materials taking into account their peculiarities, including composition and particle size. Concerning *in vivo* studies, there are just a few studies reported in the literature. Moreover, those *in vivo* studies involved the use of rats or mice and also zebrafish (Ruyra et al., 2015; Liu et al., 2017; Raju et al., 2020; Zhu et al., 2020). These models are indeed useful but presents issues such as reproducibility, costs, and ethics approval, among others.

The aim of this study is to demonstrate the suitability of Amphipods for the development of a fast, low cost, and reliable assay, to evaluate the *in vivo* toxicity of MOFs. Thus, six different MOFs were synthesized, and their *in vitro* cytotoxicity was first assessed using a colorimetric assay and a macrophage cell line. These results were used with comparison purposes to validate the *in vivo* toxicity tests performed using Amphipods and increasing concentrations of the different MOFs.

## Materials and Methods

### Synthesis of MOFs

The MOFs were synthesized by the solvothermal method and following procedures previously reported in the literature. The general protocol includes dissolving the inorganic salt of the metal and the corresponding organic ligand in an adequate solvent, together with a modulator when necessary. The mixture is then placed in a 15 mL solvothermal reactor of Teflon, which is then introduced in a stainless-steel autoclave (Parr Instrument Company, Moline, IL, USA). The reactor is placed in a Memmert oven model UF30 (Schwabach, Germany) for a fixed time and at a specific temperature depending on the MOF. Once the synthesis is finished, the MOF powder is separated from the solution by filtration and washed several times with different solvents to remove unreacted reagents and to exchange the guest solvent molecules in the pores using a solvent with lower boiling point. Finally, the MOF is activated by placing the powder in the oven at temperatures higher than the boiling point of the washing solvent to evacuate the pores. Table 1 includes the composition of the tested MOFs, together with their structure and main synthetic conditions (Furukawa et al., 2014; Mostakim et al., 2015; Trickett et al., 2015; Yao et al., 2016; Reinsch et al., 2018; González-Hernández et al., 2019).

**Table 1:** Composition, structure and synthetic conditions for the MOFs tested in the present study.

**MOF**	**Metal**	**Organic ligand**	**Solvent/Modulator**	**Temperature (°C)/time (h)**	**Structure**	
UiO-64	Zr (IV)	2-butenedioic acid	DMF/HCl	150°C/24 h	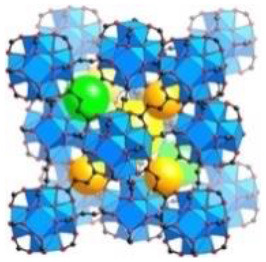	Adapted with permission from Furukawa et al. (2014) Copyright (2014) American Chemical Society.
UiO-66	Zr (IV)	benzene-1,4-dicarboxylic acid	DMF/HCl	150°C/24 h	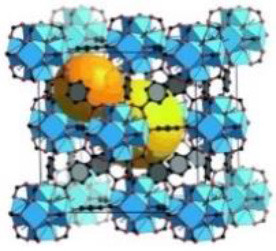	Adapted with permission from Trickett et al. (2015) Copyright (2015) John Wiley & Sons.
CIM-80	Al (III)	2-methyl-2-butenedioic acid	water/urea	150°C/3 h	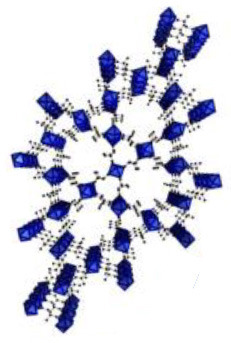	Adapted with permission from Reinsch et al. (2018) Copyright (2018) John Wiley & Sons.
CIM-81	Zn (II)	1,2,4-triazole & benzene-1,4-dicarboxylic acid	DMA/–	120°C/72 h	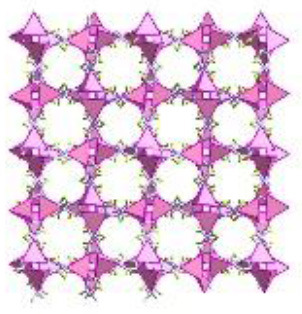	Adapted with permission from González-Hernández et al. (2019). Copyright (2019) MDPI.
CIM-91	Zn (II)	1,2,4-triazole & benzene-1,4-dicarboxylic acid	DMF/–	120°C/72 h	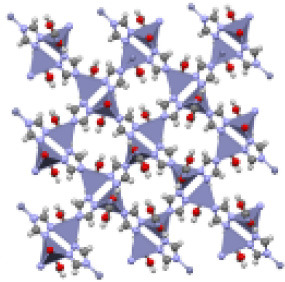	Obtained using Mercury 4.3.0 and crystallographic data from Yao et al. (2016)
CIM-84	Zn (II)	2-methyl-2-butenedioic acid	DMF/acetic acid	120°C/48 h	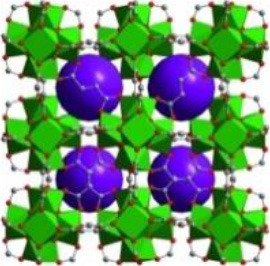	Reproduced with permission from Mostakim et al. (2015) Copyright (2015) John Wiley & Sons.

The MOF UiO-64(Zr) (also known as MOF-801) was prepared according to the protocol described by Furukawa et al. (2014). Briefly, 1 mmol of ZrCl_4_ (≥99.5%, Sigma-Aldrich, Steinheim, Germany) and 1.5 mmol of 2-butenedioic acid (98%, Sigma-Aldrich) were mixed in 15 mL of *N, N*-dimethylformamide (DMF, 99.5%, Merck, Darmstad, Germany), using 1 mL of HCl (37% v/v, Scharlau, Barcelona, Spain) as modulator. The synthesis was carried out at 150°C for 24 h. The MOF was washed with 15 mL of DMF and 30 mL of methanol (>99%, Panreac AppliChem, Barcelona, Spain), and then activated at 150°C for 24 h.

The protocol for the synthesis of UiO-66(Zr) was very similar (Katz et al., 2013). One millimole of ZrCl_4_, 1.5 mmol of benzene-1,4-dicarboxylic acid (98%, Sigma-Aldrich) and 1 mL of HCl were dissolved in 15 mL of DMF. The conditions for the synthesis, washing and activation steps were the same as for UiO-64.

The MOF CIM-80(Al) was prepared following the procedure reported by Rocío-Bautista et al. (2018). One millimole of Al(NO_3_)_3_·9H_2_O (≥98%, Sigma-Aldrich) and 1 mmol of 2-methyl-2-butenedioic acid (99%, Sigma-Aldrich) were dissolved in 15 mL of ultrapure water (obtained from a Milli-Q water purification system, Watford, UK), together with 0.5 mmol of urea (99.5%, Sigma-Aldrich) as modulator. The synthesis of the MOF was accomplished at 150°C for 3 h. The resulting MOF was washed with 15 mL of water and activated at 150°C for 24 h.

The MOFs CIM-81(Zn) and CIM-91(Zn) (also known as FJU-40-H) containing mixed ligands were synthesized according to González-Hernández et al. (2019) and Yao et al. (2016) with slight modifications, respectively. Two millimole of Zn(NO_3_)_2_·6H_2_O (98%, Sigma-Aldrich), 2 mmol of 1,2,4-triazole (98%, Sigma-Aldrich) and 1 mmol of benzene-1,4-dicarboxylic acid were dissolved in 15 mL of *N, N*-dimethylacetamide (DMA, ≥99%, Merck) for CIM-81(Zn), and in 15 mL of DMF for the preparation of CIM-91(Zn). In both cases, the mixture was heated at 120°C for 72 h to obtain the MOFs. Then, they were washed with 15 mL of DMA or DMF and 30 mL of acetone (>99%, Panreac AppliChem). The activation protocol consisted in stirring the MOF powder in 15 mL of acetone for 24 h (twice) followed by activation at 100°C for 24 h.

In the case of CIM-84(Zr), the synthetic protocol reported by Mostakim et al. (2015) was slightly modified. One millimole of ZrCl_4_ and 1 mmol of 2-methyl-2-butenedioic acid were mixed in 4 mL of DMF using 2 mL of acetic acid (≥99.8%, Honeywell, Seelze, Germany) as modulator. The reaction was accomplished at 120°C for 48 h. The MOF powder was washed with 15 mL of DMF and 30 mL of acetone. For the activation, the MOF was kept in 15 mL of acetone under agitation for 24 h (twice) and then dried at 100°C for 24 h.

The powder X-ray diffraction pattern (PXRD) was obtained for each MOF and compared with the respective theoretical pattern to verify their correct preparation, as shown in Supplementary Figure 1. An Empyrean diffractometer (PANalytical, Netherlands) operating with Bragg-Brentano geometry and using Cu-Kα radiation (λ = 1.5418 Å) over the angular range 5.00–80.00° for 10 min was used to obtain the PXRD patterns. Moreover, Supplementary Figures 2, 3 include the thermogravimetric curves and N_2_ adsorption isotherms obtained for several of the synthesized MOFs as representative examples, showing their thermal stability and experimental surface area. The thermogravimetric analysis (TGA) was carried out using a Perkin Elmer Pyris Diamond TGA equipment (Waltham, MA, USA), while the adsorption data were obtained using a V2365 Gemini Surface Area Analyzer from Micromeritics (Norcross, GA, USA) at 77 K. The surface area shown in Supplementary Figure 3 was calculated by the Brunauer, Emmett, and Teller (BET) method with a single point reference. UiO-64(Zr) and CIM-81(Zn) MOFs were also characterized by Scanning Electron Microscopy (SEM) using an EVO 15 microscope (ZEISS, Germany) equipped with a 50 mm^2^ silicon drift X-MAX detector (Oxford Instruments, Abingdon, UK). The SEM images are also included in Supplementary Figure 1.

### Cells and Amphipods

The J774A.1 murine macrophage cell line (ATCC TIB-67) from LGC Promochem (Barcelona, Spain) was used for the *in vitro* cytotoxicity tests. They were cultivated in Dulbecco's Modified Eagle Medium (DMEM) containing 10% of fetal calf serum and 10 μg/mL of gentamicin (Gibco Life Technologies, Madrid, Spain). *Gammaropsis atlantica* amphipods were collected from Punta del Hidalgo, Tenerife (Spain) and were cultured in the laboratory in filtered sea water collected from the same area. These amphipods were first washed three times with sea water and then cultured in 24 well plates at 28°C, being then kept until further experiments were performed.

### *In vitro* Cytotoxicity Assay Using Macrophages

The *in vitro* cytotoxicity of the MOFs was evaluated using the AlamarBlue® cell viability assay as previously described (Sifaoui et al., 2017). Briefly, the cells were seeded on a Nunc® 96-well plate (ThermoFisher Scientific, Madrid, Spain) with 50 μL of a stock solution containing 2·10^5^ cells/mL in RPMI without phenol red (Roswell Park Memorial Institute, Thermo Fisher Scientific Inc., Waltham, MA, USA). A countess II FL automatic cell counter (Thermo Fisher Scientific, Madrid, Spain) and Trypan Blue (Thermo Fisher Scientific, Madrid, Spain) were used for determining the cell density. Then, 50 μL of MOF suspension in distilled water at different increasing concentrations ranging from ~5 to 0.04 mg/mL were added to the wells. Finally, the Alamar Blue® reagent (Life Technologies, Madrid, Spain) was added into each well at 10% of the total well volume (100 μL), and the plates were incubated for 24 h at 37°C. Negative controls were prepared by adding 50 μL of RMPI media instead of the MOF suspension.

The emitted fluorescence was measured with an EnSpire® Multimode Plate Reader (Perkin Elmer, Madrid, Spain) at 570/585 nm. Dose-response curves were plotted, a linear regression analysis with 95% confidence limits was performed, and the CC_50_ values were calculated. Experiments were performed in triplicate.

### Statistical Analysis

All assays were carried out in triplicate. The results were defined as the mean values of three experiments. The obtained inhibition curves were performed using the Sigma Plot 12.0 software program (Systat Software Inc.). Statistical analyses were performed using the GraphPad Prism 8.0.2. Differences between the values were assessed using a one-way analysis of variance (ANOVA). Data are presented as means ± SD, and *p* < 0.0001 was considered statistically significant.

### *In vivo* Toxicity Assay Using Amphipods

The *in vivo* assay was performed according to the standard protocol UNE-EN ISO 6341:2013 for the determination of the inhibition of the mobility of *Daphnia magna* Straus for the assessment of water quality. Briefly, Nunc® 24 Deep-Well plates were filled with 1 mL of filtered seawater and from 4 to 6 amphipods individuals were added to each well. Then, a certain volume of a suspension of the MOFs, which was prepared by dispersing the MOF powder in filtered seawater, was added to the wells to obtain the desired concentration. The plates were kept at 20°C under a slight agitation. The mobility of the amphipods was evaluated after 24 h of exposure, while the count of dead and alive individuals was carried out after 48 h of exposure taking into account their color, which turns from grayish brown to pinkish orange when they die. In the preliminary test to estimate the range of MOF concentration to perform the assay, the MOFs were added in a concentration of ~5 mg/mL. The definitive assay was carried out in triplicate by adding the MOFs at ~2.5 and ~5 mg/mL. In all the experiments, 3 controls groups were included, in which the amphipods were not in contact with any additional substance apart from seawater.

## Results and Discussion

### *In vitro* Cytotoxicity of MOFs

**Figure 1:**
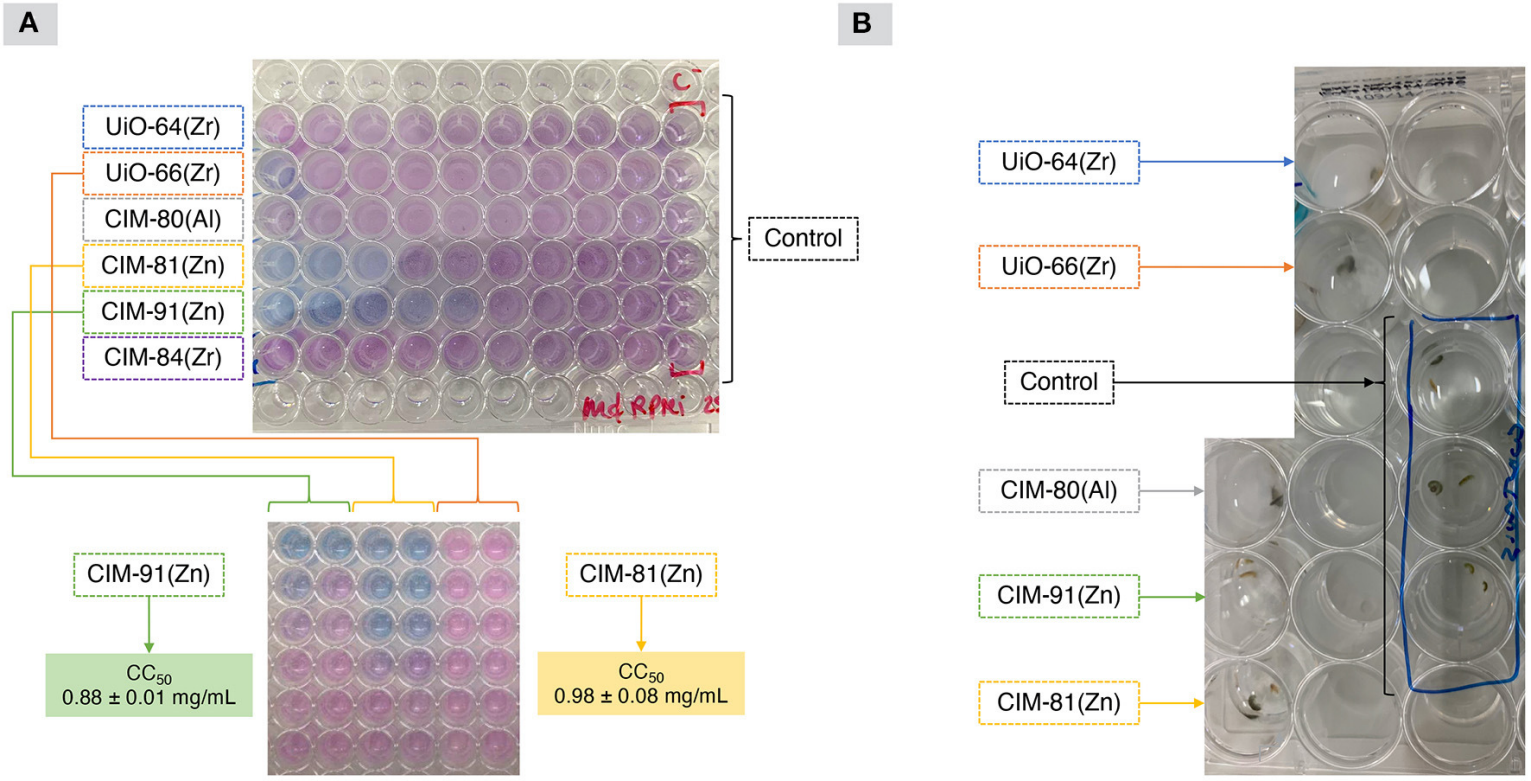
**(A)** Results obtained from the *in vitro* cytotoxicity tests using macrophages and, **(B)** representative image of the *in vivo* toxicity assay using amphipods.

The *in vitro* cytotoxicity of MOFs was conducted on murine macrophages J774.A1. Among the six tested MOFs, UiO-64(Zr), CIM-80(Al) and CIM-84(Zr) were not toxic toward the tested mammal cell line, with a LC_50_ > 5 mg/mL as shown in Table 2. The remaining MOFs seemed toxic after visual evaluation of the well plates after the incubation time, as it is observed in Figure 1A. The test was repeated for these MOFs, which confirmed the non-toxicity of UiO-66(Zr), while CIM-81(Zn) and CIM-91(Zn) exhibited low to high toxicity with a LC_50_ < 1 mg/mL as shown in Table 2. Those MOFs are composed of Zn(II) and a mixture of terephthalic acid and 1,2,4-triazole as organic linkers. It has been reported by Ren et al. (2014) that the toxicity in rat pheochromocytoma PC12 cells may be due to the release of Zn(II) into the cytosol: it may affect the cell metabolism since it disrupted cellular zinc. Moreover, these particular MOFs, CIM-81(Zn) and CIM-91(Zn), present a larger crystal size in comparison with other MOFs as it can be observed in Supplementary Figure 1. This characteristic may be the main responsible of the results obtained in this assay since the big particle size could smash the cells and cause an irreversible physical damage. Therefore, the particle size is a really important factor to take into account when studying the toxicity of MOFs. The tested MOFs containing Zr(IV) and Al(III) as metal unit did not present toxicity toward the murine macrophages, which could due to the non-toxicity of Al(III) and the biocompatibility of Zr(IV). Indeed, several works have confirmed and reported the diverse uses of the latter in biomedical applications (von Recum, 1998) Besides, the non-cytotoxicity of the MOF CIM-80(Al) toward murine macrophages had been already reported (Rocío-Bautista et al., 2018).

**Table 2:** LC_50_ corresponding to the *in vitro* cytotoxicity assay for different MOFs using murine macrophages J774.A1 after 24 h of incubation.

**MOF**	**LC_50_ mg/mL**
UiO-64(Zr)	>5
UiO-66(Zr)	>5
CIM-80(Al)	>5
CIM-81(Zn)	0.98 ± 0.08
CIM-91(Zn)	0.88 ± 0.01
CIM-84(Zr)	>5

### *In vivo* Toxicity of MOFs

The amphipods were cultured in the mentioned conditions showing a resistance of more than 10 days incubated in filtered seawater. When toxicity (*in vivo*) was tested by incubating amphipods with different concentrations of the MOFs included in this study (as shown in Figure 1B and Table 3), it was observed that only CIM-80(Al) and CIM-84(Zr) did not show any toxicity at the different tested concentrations. Moreover, the animals incubated with these MOFs showed same conditions as the ones observed in the controls. Since amphipods are very active organisms, an extra factor was checked: their motility. In the case of UiO-66(Zr)-incubated amphipods, all of them were alive but with reduced mobility. Thus, this fact was added as an extra sign of less toxicity in this assay in comparison with the remaining MOFs, which were toxic and caused the death of the animals in 48 h. The obtained results were performed twice in triplicate, obtaining the same data each time.

**Table 3:** Results from the *in vivo* cytotoxicity assay for different MOFs using amphipods after an exposure time of 48 h.

	**Preliminary test**	**Definitive test**	
**MOF**	**5.0 mg/mL**	**2.5 mg/mL**	**5.0 mg/mL**
UiO-64(Zr)	One alive	One alive	One alive
UiO-66(Zr)	Alive with reduced mobility	Alive with reduced mobility	Alive with reduced mobility
CIM-80(Al)	All alive	All alive	All alive
CIM-81(Zn)	All dead	All dead	All dead
CIM-91(Zn)	All dead	All dead	All dead
CIM-84(Zr)	All alive	All alive	All alive
Control (×3)	All alive	All alive	All alive

It is important to highlight the similarities between the results obtained with both *in vitro* and *in vivo* assays. CIM-81(Zn) and CIM-91(Zn) induced the death of all the animals, maybe due to the release of Zn(II) and its effect on some biological-metabolic processes as it was discussed in the *in vitro* assay section. In the case of UiO-64(Zr), UiO-66(Zr) and CIM-84(Zr), despite all of them contain the biocompatible metal Zr(IV), the higher toxicity of the UiO-type MOFs in this assay may be related to their smaller particle size (around 100 nm) in comparison with CIM-84(Zr) when synthesized under these conditions (Cho et al., 2019; Marshall et al., 2019), which may favor their penetration in the organisms. Therefore, the non-toxicity of CIM-80(Al) and CIM-84(Zr) could be associated to the low toxicity of their metal component and their relatively larger particle size.

These results also agree with other studies reported in the literature dealing with zebrafish (Ruyra et al., 2015). Thus, several nano-MOFs containing Zn(II) and imidazolate-based organic linkers (ZIF-7 and ZIF-8, specifically) caused a significant decrease in the embryo survival, while nano-sized UiO-66(Zr) MOF provoked significant damages to the yolk sac. Other MOFs containing Mg(II) and Fe(II) showed negligible toxicity. The findings from that study suggested that the main source of MOF toxicity is the release of metals because of their degradation, which leads to the formation of other species with harmful effects. In a similar study, Liu et al. evaluated the toxicity of several MOFs containing Zr(IV) metal ions toward zebrafish as a preliminary study for their latter application in imaging therapy (Liu et al., 2017). In this case, there were not lesions during the growth of the zebra fish, but the MOFs were accumulated in the intestine and yolk sac. Indeed, other organisms like Amphipods have been already reported in the literature with the aim of evaluating the toxicity of MOFs. In a previous study (Raju et al., 2020), *Artemia salina* (brine shrimp) were used to evaluate the toxicity of different concentrations of Ni-MOF [Ni(II) + 2-methylimidazole] in seawater. However, lower concentrations of MOFs were tested in comparison with the present study.

All other previous studies using *in vivo* assays to evaluate the toxicity of MOFs have been mainly focused on the use of zebrafish models, while a few of them used mice or rats (Ruyra et al., 2015; Liu et al., 2017; Raju et al., 2020; Zhu et al., 2020). Zebrafish has become a very important tool to assess the toxicity of several chemicals due to their small size, which allows conducting numerous analysis and repetitions and reduces the costs. They possess high fecundity and fast development, which reduces the analysis time and leads to more data to obtain more statistically reliable results. Moreover, they are very well characterized so it is easier the understanding of the toxic effects of the tested substances. Therefore, they are a promising alternative to toxicity assessments based on mammalians. Nevertheless, protocols using zebrafish need to be further standardized, they require ethics approval and their breeding may become a high-cost process as it happens when using rodents (Ruyra et al., 2015; Jia et al., 2019; Liu et al., 2019; Raju et al., 2020). Amphipods also comply with all the mentioned advantages but they do not require the need of ethics approval and also are less expensive to keep than the mentioned *in vivo* models above, including zebrafish, mice and rats. Nevertheless, it is important to highlight that differences between field amphipods and laboratory-cultured amphipods have been previously reported (Menchaca et al., 2010; Boets et al., 2012). Thus, laboratory cultured animals showed much lower sensitivity than field collected ones. Therefore, for optimal conditions, field amphipods should be used in toxicity assays, which is the case of the present study. Considering these characteristics and the reliable results found when using amphipods in the present study, they can be considered a potential high-throughput toxicity screening method to evaluate the toxicity of numerous materials and chemicals.

## Conclusions

The developed *in vivo* model using amphipods was shown to be a reliable, easy to perform, and low-cost platform to check for MOFs toxicity as well as for other solid crystalline materials. Given the potential of this simple method, we propose the standardization of this assay for the evaluation of biocompatibility of reticular materials, and also as an alternative *in vivo* assay for toxicity in therapeutics among others. Nevertheless, further studies including field and laboratory cultured animals as well as amphipods collected in the wild in different seasons (to avoid possible sensitivity variations) should be included in future assays.

## Data Availability Statement

The original contributions presented in the study are included in the article/Supplementary Material, further inquiries can be directed to the corresponding author/s.

## Author Contributions

IS and IP-F contributed to design and performance of assays. JL-M, JP, and VP contributed to conception and design of the study. IS, VP, IP-F, and JL-M wrote the first draft of the manuscript. All authors organized the database, performed statistical analysis, contributed to manuscript revision, read and approved the submitted version.

## Conflict of Interest

The authors declare that the research was conducted in the absence of any commercial or financial relationships that could be construed as a potential conflict of interest.
