# Supplementary material for: A Simple in vivo Assay Using Amphipods for the Evaluation of Potential Biocompatible Metal-Organic Frameworks
Source: Front Bioeng Biotechnol. 2021 Feb 1;9:584115. doi: 10.3389/fbioe.2021.584115 (PMC7882682; doi:10.3389/fbioe.2021.584115)
Supplement: Supplementary file 1 [file Data_Sheet_1.PDF]

Supplementary Material

1 Supplementary Figures

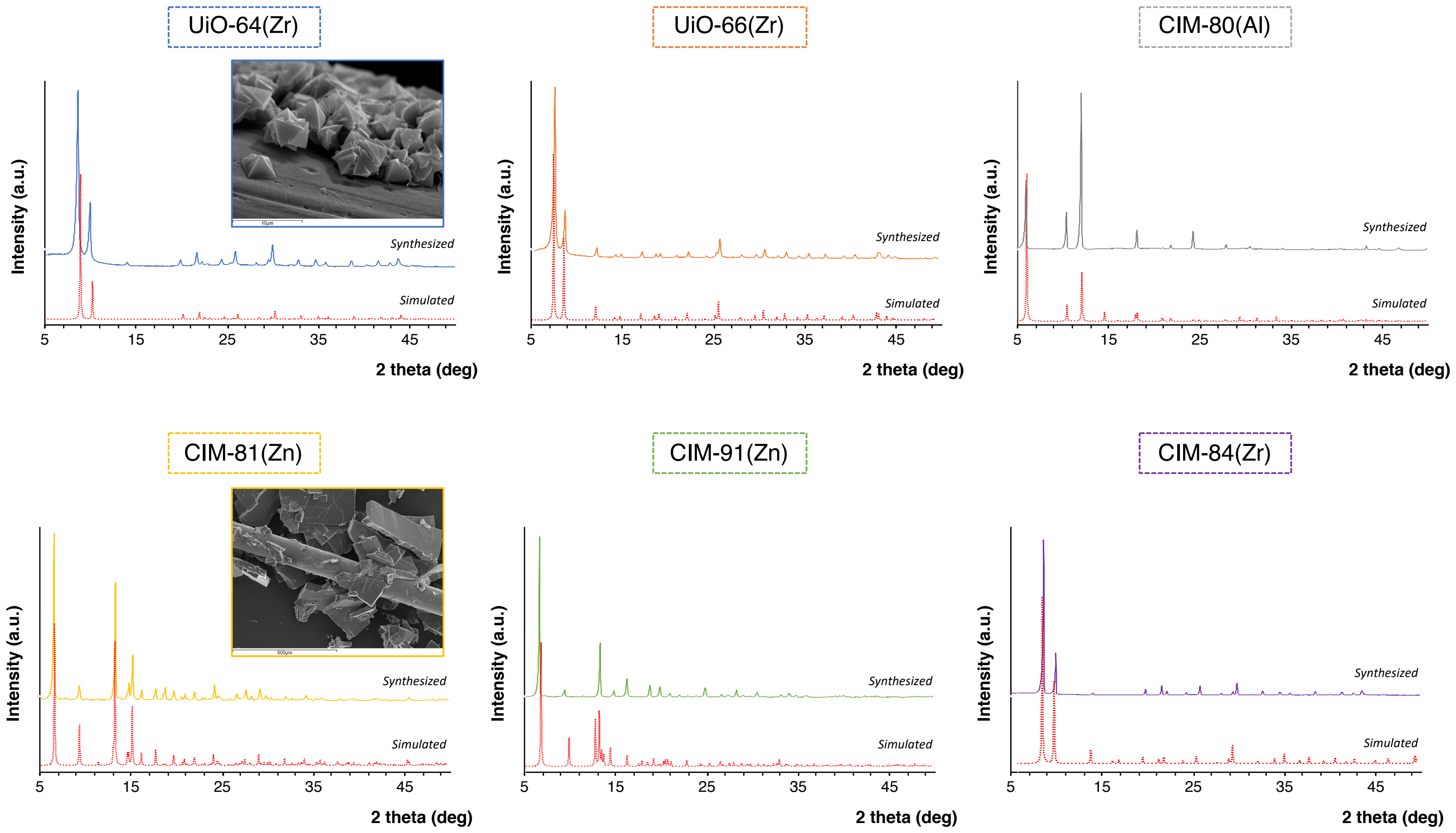

Figure S1. PXRD patterns for the synthesized MOFs after their activation, together with the theoretical patterns obtained from the Cambridge Crystallographic Data Centre.

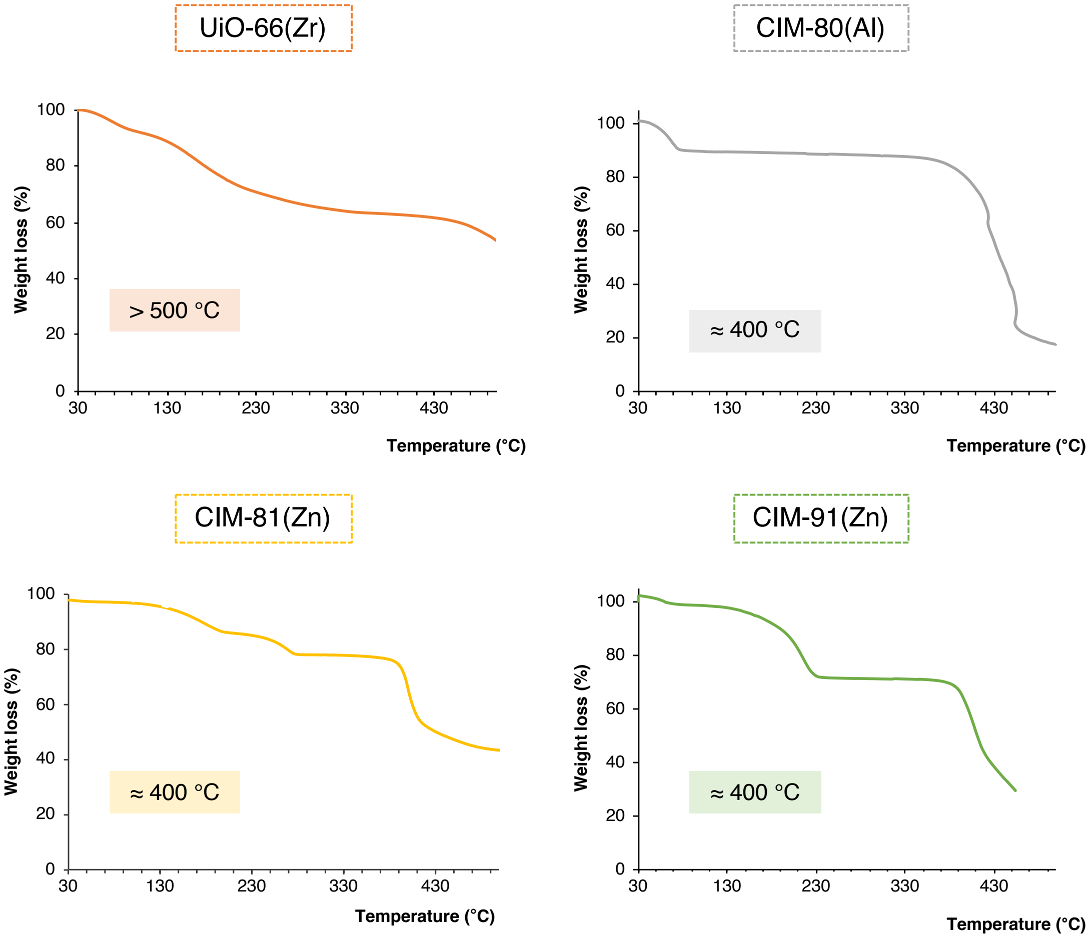

Figure S2. Thermogravimetric curves showing the thermal stability of several of the synthesized MOFs as representative examples, including the approximate temperature at which decomposition starts.

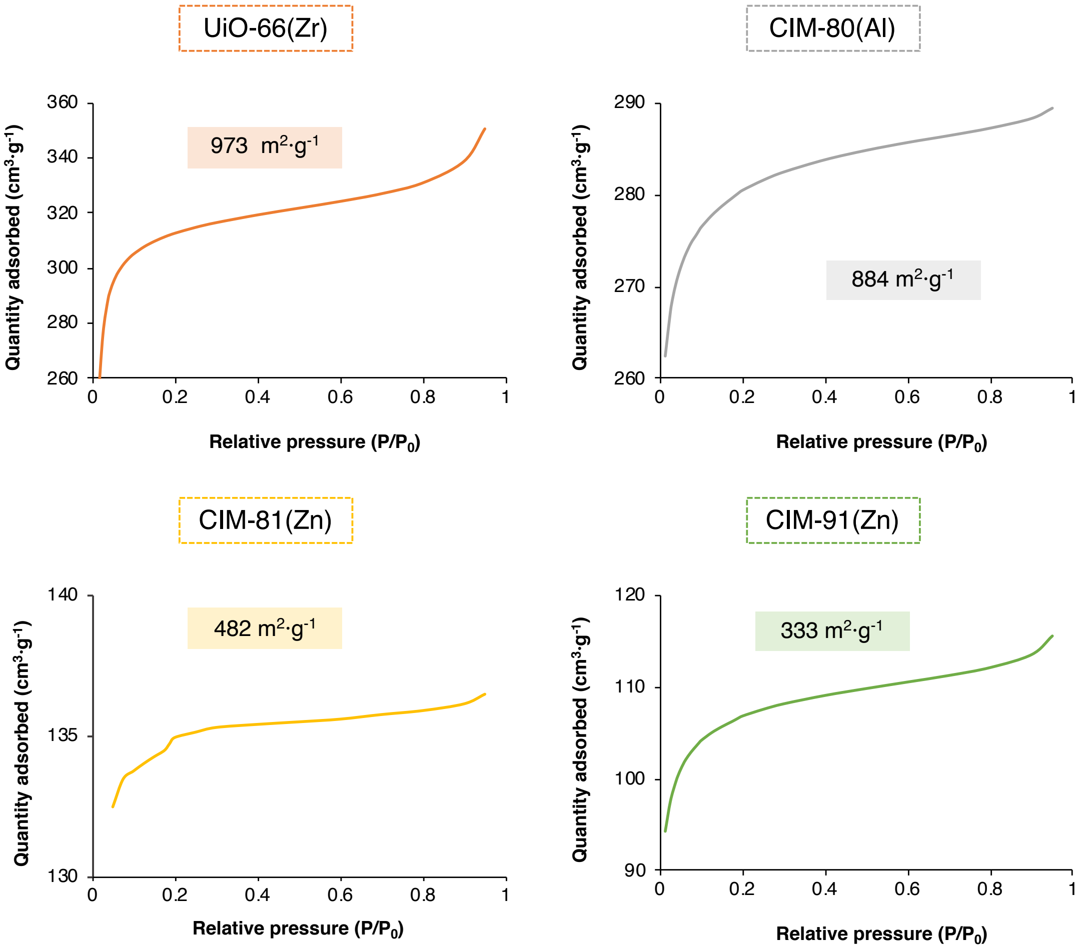

Figure S3. N_2 adsorption isotherms of several of the synthesized MOFs as representative examples, together with the Brunauer-Emmett-Teller (BET) surface area value obtained.
